# “I want it all”: exploring the relationship between entrepreneurs’ satisfaction with work–life balance, well-being, flow and firm growth

**DOI:** 10.1007/s11846-023-00623-2

**Published:** 2023-03-02

**Authors:** Mateja Drnovšek, Alenka Slavec, Darija Aleksić

**Affiliations:** School of Economics and Business, University of Ljubljana, Kardeljeva Pl. 17, 1000 Ljubljana, Slovenia

**Keywords:** Satisfaction with work–life balance, Well-being, Flow at work, Firm growth, L25, L26, I31

## Abstract

Drawing from the conservation of resources theory, we explore how two personal resources (satisfaction with work–life balance and experience of flow at work) contribute to two important outcomes in entrepreneurship: entrepreneurs’ subjective well-being and firm growth. Although previous research has emphasized the importance of personal factors for firm growth and explored a variety of factors affecting entrepreneurs’ subjective well-being, little attention has been paid to the role of satisfaction with work–life balance as a critical personal resource for entrepreneurs. With this study, we find that entrepreneurs’ satisfaction with work–life balance is positively associated with subjective well-being, which, in turn, mediates the relationship between satisfaction with work–life balance and firm growth. Our study also shows that experiencing flow at work accentuates the relationship between satisfaction with work–life balance and subjective well-being. Based on our findings, we offer implications for practicing entrepreneurs in terms of how to achieve higher levels of well-being and better firm growth. Specifically, we emphasize the benefits of achieving satisfaction with work–life balance, as this is important for an entrepreneur’s subjective well-being and has an indirect impact on firm growth. Stakeholders in entrepreneurial ecosystems should embrace subjective well-being as an important indicator of firm outcomes alongside traditional economic measures.

**Supplementary Information:**

The online version contains supplementary material available at 10.1007/s11846-023-00623-2.

## Introduction

Entrepreneurs constitute a major group in the global workforce, contributing to economic growth (Davidsson et al. 1995; Valliere and Peterson 2009), job creation (Ayyagari et al. 2003; Deijl et al. 2013), value added (Julien and Ramangalahy 2003) and innovation (Hall et al. 2009; Fernandes and Ferreira 2021). Along with these important outcomes for flourishing national economies, evidence from labor economics suggests that entrepreneurs also tend to experience the highest levels of well-being in the workforce (Naudé et al. 2014). Entrepreneurs perceive their subjective well-being as a desired outcome and a critical factor affecting their capacity to work, maintain positive relationships and live a fulfilling life (Wiklund et al. 2019; Ryff 2019). Therefore, subjective well-being is both an important personal outcome for entrepreneurs and a critical resource for attaining organizationally relevant outcomes. Specifically, previous research has associated entrepreneurs’ well-being with a range of positive outcomes, including firm performance (Stephan 2018), opportunity identification, creativity, risk-taking (Nikolaev et al. 2020) and feelings of success (Wach et al. 2016). Entrepreneurs’ well-being can be a force for positive change in society as a whole through its impact on firm growth (Wiklund et al. 2019). In entrepreneurship, firm growth is one of the most desirable organizational outcomes (Audretsch 2012) because of its important contribution by means of job creation, GDP growth and the overall well-being of national economies, among other benefits (Naudé et al. 2014; Acs et al. 2008). There is a consensus among researchers that sales growth is the best measure of growth (McDougall et al. 1992), as it reflects both short- and long-term changes in a firm, is easily obtainable, and is the most common performance indicator among entrepreneurs themselves (Davidsson et al. 2006). In this paper, we examine the dynamics of the relationship between entrepreneurs’ desired outcomes, such as subjective well-being, and firm growth. We argue that this relationship can be explained with conservation of resources (hereinafter COR) theory (Hobfoll 2001). The fundamental proposition of this theory is that individuals use their resources not only to respond to imminent stress, but also to build and conserve a reservoir of sustaining resources they expect will be needed in the future. The idea of “growth” is thus ingrained in COR theory’s core principle, namely that individuals accumulate a reservoir of resources for future needs and goals (such as firm growth).

Although COR theory has been recognized as a relevant theory to explain the gains/losses of entrepreneurs’ personal resources (Lanivich 2015), it has remained relatively silent on the role of personal resources, such as satisfaction with work–life balance, in attaining different aspects of entrepreneurs’ personal (e.g., well-being) and organizational (e.g., firm growth) outcomes. An important assumption of COR theory is that individuals seek to develop, retain and protect resources from the stressors in their environment (Hobfoll et al. 2018). Existing research has emphasized that entrepreneurs’ satisfaction with the balance between their work and family responsibilities plays an important role in managing their daily stress (Eddleston and Powell 2012), while serving as a mechanism to promote subjective well-being. Drawing from COR theory, we consider satisfaction with work–life balance to be an entrepreneur’s personal resource, since it is proximate to the self and inherent in the individual entrepreneur as a person (Hobfoll 2001). COR theory also emphasizes that resources can generate new resources in an ongoing cycle of resource accumulation known as a resource gain spiral. Furthermore, the impact of satisfaction with work–life balance as a critical resource for entrepreneurs could be augmented through the attainment of positive psychological states at work, such as flow (Mihelič and Aleksić 2017). However, to date, scholarly research has not addressed the importance of the flow experience at work in supporting entrepreneurial behaviors and outcomes, despite the fact that this experience has been seen as an important channel for reaching peak performance in entrepreneurship (Schindehutte et al. 2006).

The purpose of this paper is thus to conceptualize and empirically explore the relationships among entrepreneurs’ satisfaction with their work–life balance and experience of flow at work—which are both seen as critical personal resources for subjective well-being and as desired personal outcomes for entrepreneurs—and firm growth. In conceptualizing our research model, we build on COR theoretical ideas to provide a rich perspective on how entrepreneurs use personal resources to attain important individual and organizational outcomes, operationalized through subjective and objective indicators. We argue that personal resources (i.e., satisfaction with work–life balance and experience of flow at work) can contribute to important personal outcomes, such as an entrepreneur’s subjective well-being. In turn, subjective well-being also acts as an important personal resource for an entrepreneur, as a resource reservoir is directly related to firm growth (i.e., an organizationally relevant outcome). By turning the spotlight on entrepreneurs’ well-being as an important personal outcome, this paper contributes to research that considers not only the health of the business, but also the flourishing of the entrepreneur, since both subjective well-being and firm growth are instrumental for entrepreneurs to sustain their businesses (Gielnik et al. 2015; Shepherd and Haynie 2009; Uy et al. 2017). In the following section, we continue by developing the conceptual framework, which draws on COR theory and integrates findings from previous research in the domain of entrepreneurs’ subjective well-being, work–life balance, experience of flow at work, and firm growth. We integrate these streams of literature to develop testable hypotheses and examine them on a sample of entrepreneurs. We close with a discussion of the theoretical and practical implications of our research.

## Theoretical framework and hypotheses

### The key principles of COR theory

COR theory is a motivational theory that explains human behavior based on our evolutionary need to acquire, conserve, foster and protect resources in order to respond to strain and build a reservoir of sustaining resources for future need (Hobfoll et al. 2018). Resources are broadly defined as objects, states and conditions, and other things that people value. Although the value of resources is tied to individuals’ personal experiences and situations, and thus varies among individuals, the individual resources most valued universally are health, well-being, family, self-esteem, and a sense of purpose and meaning in life. Previous research has suggested that such resources can hold value to the extent that they are perceived to help individuals achieve their goals (Halbesleben et al. 2014).

Entrepreneurs, in particular, realize the critical nature of resources, which is why COR theory provides an informative explanation of entrepreneurial behaviors (Lanivich 2015: 864). In entrepreneurship, the most important sources of strain that can result in resource losses are tied to uncertainities surrounding the venture creation process, opportunity costs, and potential losses in the process of managing multiple roles at once, such as work and family roles (Lanivich 2015). Through the lens of COR theory, entrepreneurial behaviors can be seen as coping mechanisms for potential and actual resource loss/gain, and can be explained by key principles of the theory. First, according to COR theory, resource loss is more powerful than resource gain, with immediate effects. The primacy of resource loss suggests that resource loss will have a greater psychological impact on entrepreneurs than resource gain. The second principle from COR theory relates to resource investment, in that, entrepreneurs invest resources to protect against resource loss, recover from losses and gain resources. When resource losses are high, resource gains increase in their inherent value. Moreover, entrepreneurs with greater resources are less vulnerable to resource losses and more capable of orchestrating resource gains (Hobfoll et al. 2018). However, when entrepreneurs feel that their existing resources are exhausted, they may enter into a defensive mode to preserve the self.

In entrepreneurship, several authors have previously applied ideas from COR theory, finding that resource-induced cognitive mechanisms are important for developing and managing resources in new ventures (Dimov and Shepherd 2005), and have an impact on entrepreneurial orientation (Adomako 2021) and financial performance (Lanivich 2015). In our research, we explore how entrepreneurs use satisfaction with work–life balance and experience of flow at work as important resources to protect their valued outcomes, such as subjective well-being. In turn, resource gains at the personal level (e.g., subjective well-being) are used in a resource gain spiral to support firm-level outcomes (e.g., firm growth).

### Satisfaction with work–life balance

Satisfaction with work–life balance is defined as “achieving satisfying experiences in all life domains” (Kirchmeyer 2000: 80). However, the positive effects of work–life balance may differ for different segments of the workforce (Darcy et al. 2012). For entrepreneurs, the work and life spheres are often interconnected (Aldrich and Cliff 2003), which is why entrepreneurs may experience greater challenges in balancing their work and family lives than employees (Parasuraman and Simmers 2001). Previous research has examined these challenges from two perspectives inherent to COR theory: from the perspective of resource gains via satisfaction with work–life balance, and from the perspective of resource losses via work–life conflict (Parasuraman et al. 1996), as long working hours and risk-taking behaviors induce stress that can spill over into entrepreneurs’ personal lives (Lewin-Epstein and Yuchtman-Yaar 1991). Self-employed individuals also experience the “always-on” challenge of being readily available to their families while addressing work-related challenges (Hilbrecht and Lero 2014); this can undermine their perception of work–life balance. At the same time, entrepreneurship scholars have long acknowledged that quality of life plays an important role in explaining entrepreneurs’ desire to grow their business (Lewis 2008; Marcketti et al. 2006). Entrepreneurs’ satisfaction with their work–life balance induces positive emotions, which supports their ability to focus on using personal resources (i.e., time, effort, energy) to meet demands at work (Lanivich 2015). Entrepreneurs consider it important to achieve a satisfying balance between the quality of their personal and professional lives (Greenhaus et al. 2003; Annink and den Dulk 2012). Building on the COR literature (Hobfoll 2001), satisfaction with work–life balance represents an important resource in the resource gain spiral, which is why it comes as no surprise that entrepreneurs consider it an important goal.

### Subjective well-being of entrepreneurs

Subjective well-being relies on both hedonic and eudaimonic perspectives to explain the experiences of life satisfaction and happiness (Ryan and Deci 2001). Entrepreneurship research to date has predominantly employed the hedonic aspect of subjective well-being, which focuses on entrepreneurs’ life satisfaction as indicated by the presence of positive affect and absence of negative affect (Ryan and Deci 2001). By contrast, eudaimonic well-being is defined in terms of the degree to which a person is fully functioning, cultivates personal strengths, and contributes to the greater good (Ryan and Deci 2001). This type of well-being focuses on experiences that are objectively good for the person (Kagan 1992). In our study, we follow the hedonic idea that subjective well-being relates to an individual’s positive evaluations of his/her life, experience of pleasant emotions, high life satisfaction and fulfillment, and feeling that life is rewarding in general (Diener et al. 2002). In order to preserve their well-being, entrepreneurs are motivated to protect their existing resources (conservation principle) and acquire new resources (acquisition principle), as implied by COR theory (Marshall et al. 2020). In line with this perspective, subjective well-being reflects how entrepreneurs perceive personal resources (e.g., satisfaction with work–life balance) and how these cognitive perceptions of the environment contribute to resource gain spirals.

### Experience of flow at work

Experience of flow refers to “the holistic sensation that people feel when they act with total involvement with a sense of self-control and pleasure” (Csikszentmihalyi 1975: 7). Flow can be experienced when there is balance between the challenges inherent in a task and the skills necessary to meet those challenges. Flow is expected to be heightened when individuals see value in an activity, have clear goals and receive immediate feedback on actions (Csikszentmihalyi 1990). Experience of flow at work is characterized by absorption—total concentration and immersion in the activity; enjoyment as an outcome of cognitive and affective evaluations of the flow experience; and intrinsic motivation, which refers to the state of engagement in the activity for its own sake, rather than for external reward (Bakker 2008). In entrepreneurship, the relatively high frequency of high-intensity experiences seems to be a function of the deliberate choices made by the entrepreneur and the circumstances surrounding entrepreneurship (Shane et al. 2003). In fact, entrepreneurial job characteristics are uniquely defined by the experience of high passion, drive and spirit (Palmer et al. 2021: p.461). Specifically, it is important for entrepreneurs to have intense affective experiences in which they are immersed and to feel in complete control over their activities (Cardon et al. 2009), as this lends meaning to their identity. The attainment of fulfilling flow experiences illustrates entrepreneurs’ propensity to pursue growth by recognizing emerging opportunities (Schindehutte et al. 2006: 350) that require entrepreneurial resources. The experience of flow motivates entrepreneurs to improve and balance their skillset and the level of set challenges (Csikszentmihalyi 1997). Although the experience of flow at work consumes an individual’s time and energy, the pleasure associated with the flow experience give rise to resources, and individuals are prepared to put in effort to experience that flow again.

In the following sections, we propose hypotheses regarding the relationships between entrepreneurs’ subjective well-being, work–life balance, experience of flow at work, and firm growth.

### Direct relationship between satisfaction with work–life balance and firm growth

Previous research tells us that people who are able to achieve a satisfying balance between their professional and personal domains achieve better overall results in their work (Magnini 2009). Building on COR theory, we argue that achieving a satisfying work–life balance might also be a priority for entrepreneurs, as this represents an important personal resource that could be used in a resource gain spiral to support firm-level outcomes (e.g., firm growth). Existing research also suggests that maintaining a good balance between work and personal life plays an important role in explaining the entrepreneurial desire to grow one’s business (Lewis 2008; Marcketti et al. 2006; Khanin et al. 2021). Delmar and Wiklund (2008) found that entrepreneurs who feel that their work is not interfering with or diminishing the quality of their personal life are more motivated, which in turn affects important firm outcomes such as growth. Overall, a greater balance between work and non-work roles can improve entrepreneurs’ commitment and willingness to grow their business (Eddleston and Powell 2012). In line with this reasoning:

#### H1

We expect a positive association among entrepreneurs’ level of satisfaction with their work–life balance and firm growth.

### The mediation effects of an entrepreneur’s well-being

Previous research suggests that satisfaction with work–life balance promotes an individual’s overall well-being (Greenhaus et al. 2003). The positive impact of satisfaction with work–life balance on subjective well-being takes several pathways. First, the involvement of entrepreneurs in multiple roles protects them from the effects of negative experiences in any one role (Barnett and Hyde 2001). Furthermore, entrepreneurs who are satisfied with their work–life balance are “primed to seize the moment” when confronted with a role demand because no role is seen as “less worthy of one’s alertness than any other” (Marks and MacDermid 1996: 421). Accordingly, a satisfying balance between work and life arenas leads to higher subjective well-being because balanced individuals experience lower levels of stress and greater role ease—both of which are associated with greater well-being (Frone et al. 1992; Greenhaus et al. 2003). Some authors have provided empirical evidence that work-related well-being spills over to become context-free well-being (Hakanen and Schaufeli 2012). Specifically, the autonomy of an entrepreneurship career enables entrepreneurs to meet the dual demands of work and non-work domains, experience less family–work conflict (i.e., less loss of resources) and, thus, consistent with COR theory, experience higher life satisfaction (Parasuraman et al. 1996) (i.e., gain of resources at the personal level). Therefore,

#### H2a

We expect a positive association between entrepreneurs’ level of satisfaction with work–life balance and their well-being.

Drawing from previous research evidence and COR theory, we also expect that entrepreneurs’ subjective well-being is related to firm growth. Consistent with COR theory, we argue that well-being, which has an essential positive affective component (Diener et al. 2002), serves as a fundamental human resource that is critical to thriving. Specifically, positive affective states, such as the experience of well-being, build enduring personal resources (Lee et al. 2020), which can, in turn, have a positive impact on organizationally relevant outcomes such as firm growth. In the entrepreneurship literature, well-being is seen as “a valuable variable in its own right” (Shepherd and Haynie (2009: 330), and previous studies have also pointed to the direct effects of well-being on firm performance (Dijkhuizen et al. 2018), firm goals (Uy et al. 2017) and proactive venture behaviors (Foo et al. 2009). In line with this reasoning:

#### H2b

We expect a positive association between entrepreneurs’ well-being and firm growth.

Furthermore, beyond the direct effects of subjective well-being on firm growth, we also expect that subjective well-being functions as an intermediary between the effects of personal resources, such as satisfaction with work–life balance, and important outcomes, such as firm growth (Hobfoll et al. 2018). We argue that through the resource gain spiral associated with subjective well-being, resources related to satisfaction with work–life balance exert an influence on firm growth. Entrepreneurs who are satisfied with their work–life balance are likely to experience higher subjective well-being as an outcome of their successful navigation of personal and professional roles, and therefore experience higher firm growth. However, when entrepreneurs experience challenges to their work–life balance, they must use resources to achieve that balance. Achieving work-life balance will have immediate effects on the entrepreneur’s well-being, but not on firm growth outcomes. If the work–life imbalance persists, resources associated with well-being will be compromised, which will eventually have effects on firm growth. In other words, the direct effect of well-being on firm growth does not provide the complete picture of how well-being as a resource contributes to firm growth. To gain the full perspective, one must also account for the intermediary role of well-being as a resource, as well-being can have a positive impact on firm performance (Baron 2007) by recharging entrepreneurs’ psychological resources, such as optimism, resilience and self-esteem, and by energizing entrepreneurs to persist despite challenges that others would consider impossible to overcome (Foo et al. 2009). This perspective is aligned with the agentic roles that entrepreneurs play in developing and engaging resources to dynamically interact with external enablers in the environment (Markman and Baron 2003; Davidsson et al. 2020; Kimjeon and Davidsson 2021). In fact, subjective well-being can also be seen as a function of “one’s commitment to the valued future and enablement to take steps to realize it” (Bandura 2011), which leads us to propose the following hypothesis:

#### H3

Entrepreneurs’ subjective well-being mediates the positive relationship between their level of satisfaction with work–life balance and firm growth.

### The moderation effect of flow experience

We argue that entrepreneurs’ affective experience of flow at work (Csikszentmihalyi 1990) strengthens the positive relationship between satisfaction with work–life balance and subjective well-being. Earlier, we pointed to three important components of the flow experience: absorption (the cognitive component), work enjoyment (the emotional component) and intrinsic work motivation (the motivational component) (Bakker 2008). The affective, cognitive and motivational mechanisms evoked by the flow experience are associated with entrepreneurs’ subjective evaluation of well-being. The affective component of the flow experience at work promotes positive emotions, such as joy and enthusiasm (Chen et al. 1999), which have adaptive benefits by building personal resources and triggering an upward spiral of emotional well-being (Fredrickson 1998). The motivational component of the flow experience at work—the intense excitement and focus—comes from being engaged in work-related entrepreneurial roles, which holds meaning for entrepreneurs’ identity (Cardon et al. 2009). The motivational energy has been found to offset the negative consequences of resource depletion (Demerouti et al. 2012) because, despite the fact that entrepreneurs may experience resource depletion, they will continue to work on activities they are intrinsically motivated to complete. The motivational energy generated through the experience of flow at work regulates the positive relationship between entrepreneurs’ satisfaction with work–life balance and their well-being. In all, flow theory expects experience of flow to have a direct impact on subjective well-being by enhancing the experience of happiness in the here and now (Moneta 2004). Furthermore, professional work has been found to be a major source of flow experiences for adults (Csikszentmihalyi and LeFevre 1989), having positive associations with well-being (Bassi et al. 2013; Peifer et al. 2020) and other organizational and personal resources (Salanova et al. 2006). In an entrepreneurship context, Sherman and colleagues (Sherman et al. 2016) found that entrepreneurs’ well-being increases in the presence of flow. This leads us to propose the regulatory (i.e., moderation) effects of the experience of flow at work:

#### H4

The experience of flow at work moderates the relationship between entrepreneurs’ satisfaction with their work–life balance and well-being such that the effects of satisfaction with work–life balance on the perceived level of subjective well-being are stronger for higher levels of flow experienced at work.

**Fig. 1:**
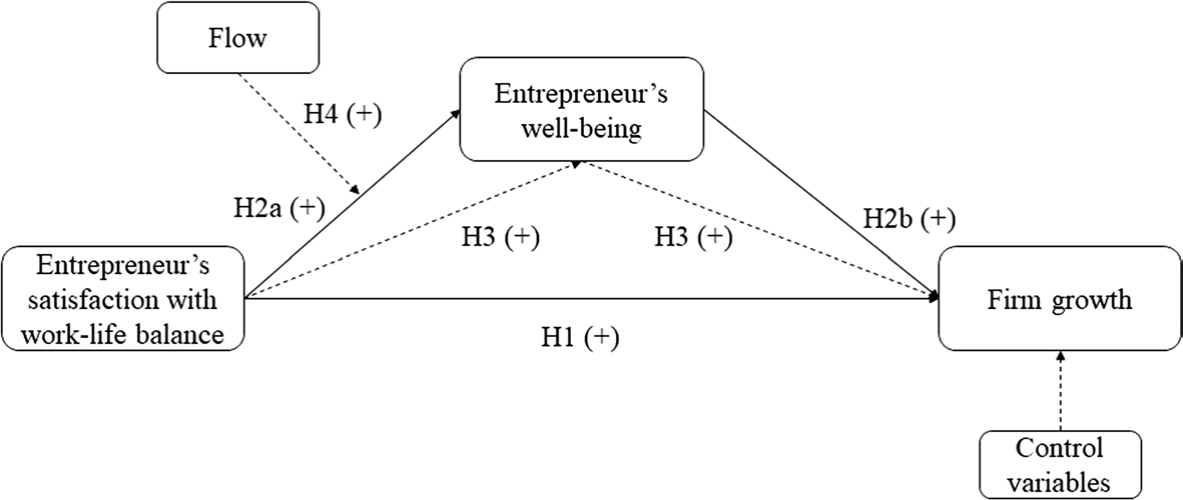
Proposed theoretical model. *Note* Dashed lines for H3 represent the mediation effect. Dashed line for H4 represents the moderation effect. Dashed line for control variable represents the control variables included in the model

In sum, our conceptual model, which draws from COR theory, proposes that satisfaction with work–life balance and experience of flow will impact important outcomes for entrepreneurs, such as subjective well-being and firm growth, because of the resource gains generated. Satisfaction with work–life balance feeds an upward cycle of resource gains, which facilitates an entrepreneur’s commitment to the desired outcomes, such as subjective well-being. Well-being, in turn, secures the entrepreneur’s commitment to valued firm outcomes, such as growth. We predict (Fig. 1) that satisfaction with work–life balance is positively related to firm growth (Hypothesis 1) and that this relationship is mediated by subjective well-being (Hypothesis 3). We also hypothesize that flow at work moderates the relationship between satisfaction with work–life balance and subjective well-being such that the relationship between satisfaction with work–life balance and subjective well-being will be even stronger for higher levels of flow at work (Hypothesis 4). These relationships are controlled by the entrepreneur’s personal characteristics and the characteristics of the firm.

## Data collection, measurement scales and summary statistics 

The empirical part of our research is based on quantitative data collected in 2017 from a sample of entrepreneurs in Slovenia. We collected the data via an online questionnaire sent to privately owned firms with 5 to 249 employees. Following the recommendations of Dillman et al. (2009), we gathered 140 usable responses, yielding a 5.71 percent response rate. We coupled responses with objective financial data from financial reports published between 2017 and 2020, where available. The final data sample consisted of 115 cases. In the sample, 22 percent of the entrepreneurs were female; on average, entrepreneurs were 52 years old and had 28 years of working experience. The majority of the firms were small (10–49 employees; 50 percent), 32 percent had up to nine employees, and 18 percent had 50–249 employees. Based on NACE classification, the three industries most represented among the sampled firms were manufacturing (28 percent), wholesale and retail trade (22 percent), and professional services (15 percent).

### Measurement scales

Respondents assessed all survey items on a 5-point Likert scale. We report scale items along with factor loadings from confirmatory factor analysis in Table 1. We applied Putrevu and Ratchford's (1997) scale to measure entrepreneurs’ satisfaction with their work–life balance. The scale’s reliability was adequate (Cronbach’s α = 0.927). Entrepreneurs’ subjective well-being was measured with a three-item measurement scale developed by Su et al. (2016). Cronbach’s α was 0.779. Experience of flow at work was evaluated using the eight-item scale by Mao et al. (2016). Three items did not load substantially on the intended factor and were omitted from the analysis. These items were: “When I engage in this activity, I feel I have clear goals,” “When I engage in this activity, I feel self-conscious” (reverse-coded), and “When I engage in this activity, I lose track of time.” The internal consistency of the scale was sufficient (Cronbach’s α = 0.696).

**Table 1:** Measurement scales and standardized factor loadings of items from confirmatory factor analysis

Scale	Factor loading
Satisfaction with work-life balance Putrevu and Ratchford (1997) (1 = Very dissatisfied to 5 = Very satisfied) I am satisfied with…	
… the way I divide my time between work and non-work life	0.807
… the way I divide my attention between work and non-work life	0.817
… how well my work life and my non-work life fit together	0.879
… my ability to balance the needs of my job with those of my non-work life	0.884
… the opportunity I have to perform my job well and yet be able to perform nonwork related duties adequately	0.860
Entrepreneur’s subjective well-being Su et al. (2016) (1 = Strongly disagree to 5 = Strongly agree)	
In general, I consider myself a very happy person	0.712
Compared to most of my peers, I consider myself more happy	0.649
I am generally very happy and enjoy life	0.832
Flow at work Mao, Roberts, Pagliaro, Csikszentmihalyi, & Bonaiuto (2016) (1 = Strongly disagree to 5 = Strongly agree) Think of an activity, which you like to do at work and mark the level of agreement about the following statements When I engage in this activity…	
I feel in control	0.507
I feel I know how well I am doing	0.413
I have a high level of concentration	0.635
I forget about personal problems	0.495
I feel fully involved	0.770
Subjective firm growth Adapted from Dijkhuizen, Gorgievski, van Veldhovena and Schalk (2018) (1 = much worse than competitors to 5 = much better than competitors) In the last three years our company has been better/worse compared to competitors in our industry at…	
… revenue growth	0.919
… growth in the number of employees	0.734
… growth in market share	0.912
Objective firm growth	
Revenue growth of each company in the period 2017–2020 divided by the average revenue growth of the industry in the same period	–

We followed the idea of using a multi-faceted measure of entrepreneurial success in measuring firm growth with subjective and objective indicators. This is similar to Dijkhuizen and colleagues’ (2018) measure of entrepreneurial success, which included both financial indicators (sales growth) and subjective indicators of success. We measured firm growth using self-reported data from the questionnaire (labeled subjective firm growth) and objective data from the official financial statements of firms (labeled objective firm growth). We asked entrepreneurs to rate on a 5-point Likert scale (1 = much worse than competitors, 5 = much better than competitors) how their firms had performed over the previous three years (2015–2017) in terms of sales growth, growth in number of employees, and growth in market share, compared to competitors. Cronbach’s α for this measure was 0.896. To measure objective firm growth, we deducted the average revenue growth of each industry in the sample between 2017 and 2020 from the absolute revenue growth of each company in the sample in the same period and standardized the score. We grouped objective firm growth into four groups based on a quartile analysis. We included four control variables: entrepreneur’s gender, age and educational level, and number of employees as an approximation of firm size. None of the control variables had a significant relationship with the dependent variables of subjective and objective firm growth. However, gender was significantly correlated with well-being, with women reporting lower well-being. 

### Summary statistics and statistical methods

We used IBM SPSS version 21.0 for descriptive statistics and reliability analyses and IBM AMOS version 21.0 for structural equation modeling. The latter technique was used to investigate the relationships among satisfaction with work–life balance, well-being, flow at work and firm growth. For analyzing the convergent and discriminant validity of the investigated constructs, we followed suggestions by Hair et al. (2010), Bagozzi and colleagues (1991) and Kim (2022). In Table 2, we report the descriptive statistics and correlations among variables for the measurement model, for which fit indices were adequate: χ^2^ = 205.956, df = 155, *p* = 0.004, CFI = 0.949, RMSEA = 0.054, and SRMR = 0.067.

**Table 2:** Descriptive statistics, correlations and squared roots of average variance extracted

Variables	Mean	SD	Min	Max	Lower bound of 95% CI	Upper bound of 95% CI	1	2	3	4	5	6	7	8
1. Well-being	3.907	0.597	1.000	5.000	3.796	4.013	**0.863**							
2. Satisfaction with work-life balance	3.757	0.727	1.000	5.000	3.631	3.890	0.696***	**0.924**						
3. Flow at work	3.792	0.509	2.400	5.000	3.694	3.887	0.460***	0.288*	**0.763**					
4. Subjective growth	3.207	0.783	1.000	5.000	3.055	3.336	0.459***	0.352***	0.241*	**0.930**				
5. Objective growth	3.000	1.420	1.000	5.000	2.739	3.261	0.145	0.010	0.194	0.170	–			
6. Gender	1.782	0.414	1	2	0.356	0.454	0.007	0.013	− 0.092	− 0.010	− 0.008	–		
7. Entrepreneur’s age	51.661	9.968	23	67	8.618	11.182	0.084	0.138	− 0.105	− 0.072	0.008	− 0.134	–	
8. Educational level	4.793	1.095	1	8	0.878	1.292	− 0.168	− 0.173	0.077	− 0.124	− 0.036	− 0.330***	0.012	–
9. Firm size	2.052	0.815	1	4	0.700	0.915	0.154	0.072	0.149	0.177	0.068	0.103	0.027	− 0.027

*N* = 115; * *p* < 0.05; ** *p* < 0.01; *** *p* < 0.001

All effects are two-tailed tests

Squared roots of average variance explained are presented in bold

CI stands for confidence interval

We applied procedural and statistical remedies following the recommendations of different scholars (e.g. Chang et al. 2010; Podsakoff et al. 2012; Williams et al. 2010) to achieve control over common method bias ex-ante and ex-post data collection and to analyze its potential presence in the dataset. We implemented the following ex-ante procedural remedies: We assured respondents’ confidentiality, developed a good cover story and instructions, pretested the questionnaire, performed a pilot study, varied the scale types and anchor labels, and labeled all scale points rather than just the end points.

For ex-post statistical remedies, we applied the common method factor technique, which showed that common method variance was 0.090. We continued with a marker variable test, testing the conceptual model with the addition of an unrelated construct. With the marker factor included, the common method variance dropped to 0.073. The fit of the model also dropped: CFI_no marker_ = 0.959 to CFI_with marker_ = 0.937, RMSEA_no marker_ = 0.048 to RMSEA_with marker_ = 0.060, SRMR_no marker_ = 0.068 to SRMR_with marker_ = 0.075. Our conceptual model also incorporates an interaction effect, which reduces the potential of common method bias, as it is more difficult for respondents to recognize the specified relationships among studied concepts (Aiken and West 1991; Harrison et al. 1996). In addition, we matched self-reported data from the questionnaire of the large-scale study with objective data from financial statements reported by firms to the national business register, giving us data from different sources. We also tested the factorial validity of the constructs by analyzing the correlations among constructs and the square root of average variance explained for each pair of constructs in the model. As shown in Table 2, we found evidence for the factorial validity of the constructs, as the square root of the average variance explained for a specific construct was higher than the correlation between the specific construct and other constructs. Based on these results, we are confident in our conclusion that common method bias was not a threat to our data.

Based on the recommendations of several scholars (Cheung and Lau 2008; Rucker et al. 2011; Hayes 2009, 2017), we performed the mediation analysis by checking the significance of indirect effects. We used a bias-corrected bootstrapping method to establish confidence intervals for the mediation and suppression effects. We performed bootstrapping on 5000 bootstrap samples at a 95 percent bias-corrected confidence level. We then mean-centered the latent variables and reduced the latent scales to single index variables in order to proceed with the moderation analysis after the mediation analysis. We introduced the interaction effect (namely, flow at work × satisfaction with work–life balance) into the mediation model following the suggestions of different scholars (e.g. Hayes 2017; Aiken and West 1991). We investigated the fit of the models following the suggestions of Hair et al. (2010), Mai et al. (2021).

## Results of the study

To test our hypotheses, we examined our survey data. Table 3 shows the results of the effect of satisfaction with work–life balance on firm growth, measured with subjective and objective indicators. We see that satisfaction with work–life balance has a significant positive relationship with the subjective indicator of firm growth (β = 0.287, *p* = 0.001). However, the results do not show a significant relationship between satisfaction with work–life balance and the objective indicator of firm growth (β = − 0.025, *p* = 0.798). These findings partially support Hypothesis 1, which predicted a positive relationship between satisfaction with work–life balance and firm growth. The fit of the model was good: χ^2^ = 86.971, df = 57, *p* = 0.006, CFI = 0.958, RMSEA = 0.068, and SRMR = 0.073.

**Table 3:** Results of hypotheses testing

Hypotheses number	Type of relation	Relation	Standardized estimate of the regression coefficient	*P* value
H1	Direct	Satisfaction with work-life balance → Subjective firm growth	0.287	0.000
		Satisfaction with work-life balance → Objective firm growth	− 0.025	0.798
	Controls	Gender → Subjective firm growth	− 0.078	0.422
		Gender → Objective firm growth	− 0.030	0.762
		Entrepreneur’s age → Subjective firm growth	− 0.119	0.195
		Entrepreneur’s age → Objective firm growth	0.006	0.952
		Educational level → Subjective firm growth	− 0.113	0.245
		Educational level → Objective firm growth	− 0.046	0.640
		Firm size → Subjective firm growth	0.162	0.078
		Firm size → Objective firm growth	0.072	0.441
H2a, H2b, H3	Direct	Satisfaction with work-life balance → Well-being	0.703	0.000
	Direct	Well-being → Subjective firm growth	0.481	0.003
		Well-being → Objective firm growth	0.299	0.066
	Direct	Satisfaction with work-life balance → Subjective firm growth	− 0.043	0.776
		Satisfaction with work-life balance → Objective firm growth	− 0.237	0.127
	Mediation	Satisfaction with work-life balance → Well-being → Subjective firm growth	0.338	0.010
		Satisfaction with work-life balance → Well-being → Objective firm growth	0.211	0.036
	Controls	Gender → Subjective firm growth	− 0.066	0.481
		Gender → Objective firm growth	− 0.023	0.814
		Entrepreneur’s age → Subjective firm growth	− 0.124	0.158
		Entrepreneur’s age → Objective firm growth	0.004	0.968
		Educational level → Subjective firm growth	− 0.080	0.388
		Educational level → Objective firm growth	− 0.028	0.778
		Firm size → Subjective firm growth	0.128	0.147
		Firm size → Objective firm growth	0.052	0.573
H4	Moderation	Flow on Satisfaction with work-life balance → Well-being	0.312	0.000
	Mediation	Satisfaction with work-life balance → Well-being → Subjective firm growth	0.144	0.030
		Satisfaction with work-life balance → Well-being → Objective firm growth	0.123	0.015
	Direct	Satisfaction with work-life balance → Well-being	0.533	0.000
	Direct	Well-being → Subjective firm growth	0.269	0.011
		Well-being → Objective firm growth	0.231	0.045
	Direct	Satisfaction with work-life balance → Subjective firm growth	0.152	0.146
		Satisfaction with work-life balance → Objective firm growth	− 0.157	0.169
	Controls	Gender → Subjective firm growth	− 0.040	0.663
		Gender → Objective firm growth	− 0.007	0.945
		Entrepreneur’s age → Subjective firm growth	− 0.116	0.168
		Entrepreneur’s age → Objective firm growth	0.017	0.857
		Educational level → Subjective firm growth	− 0.057	0.524
		Educational level → Objective firm growth	− 0.029	0.767
		Firm size → Subjective firm growth	0.158	0.061
		Firm size → Objective firm growth	0.054	0.554

Next, we tested the mediation effects of entrepreneurs’ subjective well-being in two steps. First, we investigated the relationships between satisfaction with work–life balance and subjective well-being (Hypothesis 2a) and between subjective well-being and firm growth (Hypothesis 2b). Second, we tested the mediation effect of subjective well-being on the relationship between satisfaction with work–life balance and firm growth (Hypothesis 3). As reported in Table 3, the results show that the relationship between satisfaction with work–life balance and the subjective indicator of firm growth becomes non-significant (β = − 0.043, *p* = 0.776), while the relationship between satisfaction with work–life balance and objective firm growth remains non-significant (β = − 0.237, *p* = 0.127). The results also show that satisfaction with work–life balance is positively and significantly related to subjective well-being (β = 0.703, *p* = 0.000), which supports Hypothesis 2a. Subjective well-being is, in turn, positively and significantly related to subjective firm growth (β = 0.481, *p* = 0.003), but not significantly related to objective firm growth (β = 0.299, *p* = 0.066), which means that we can only partially accept Hypothesis 2b. The fit of the data to the model was adequate: χ^2^ = 119.157, df = 92, *p* = 0.030, CFI = 0.968, RMSEA = 0.051, and SRMR = 0.069. To further investigate the indirect effects, we employed the bootstrapping technique. The results show that satisfaction with work–life balance has a significant indirect relationship with subjective firm growth (β = 0.338, *p* = 0.010) and a significant indirect relationship with objective firm growth (β = 0.211, *p* = 0.036). This provides the basis to accept Hypotheses 3—i.e., that well-being is a mediator in the relationship between satisfaction with work–life balance and firm growth in terms of subjective and objective firm growth. We report the results of the bootstrapping method in Table 4.

**Table 4:** Results for the decomposition of effects in the mediation structural equation model by means of bootstrap method

	Subjective firm growth				Objective growth			
	Un-standardized coefficient	Standard error	Standardized coefficient	*P* value (two-tailed significance)	Un-standardized coefficient	Standard error	Standardized coefficient	*P* value (two-tailed significance)
*Satisfaction with work-life balance*								
Direct effect	− 0.061	0.164	− 0.043	0.836	− 0.443	0.193	− 0.237	0.116
Indirect effect	0.480	0.154	0.338	0.010	0.394	0.165	0.211	0.036
Total effect	0.419	0.136	0.295	0.040	− 0.049	0.194	− 0.026	0.832
*Well-being*								
Direct effect	0.836	0.194	0.481	0.017	0.687	0.194	0.299	0.051
Indirect effect	0.000	0.000	0.000	–	0.000	0.000	0.000	–
Total effect	0.836	0.194	0.481	0.017	0.687	0.194	0.299	0.051

Control variables excluded from this table since their total effect on subjective firm growth and objective firm growth is presented in Table 2

Number of bootstrap samples: 5000

Bias-corrected confidence intervals: 95 BC confidence level

**Fig. 2:**
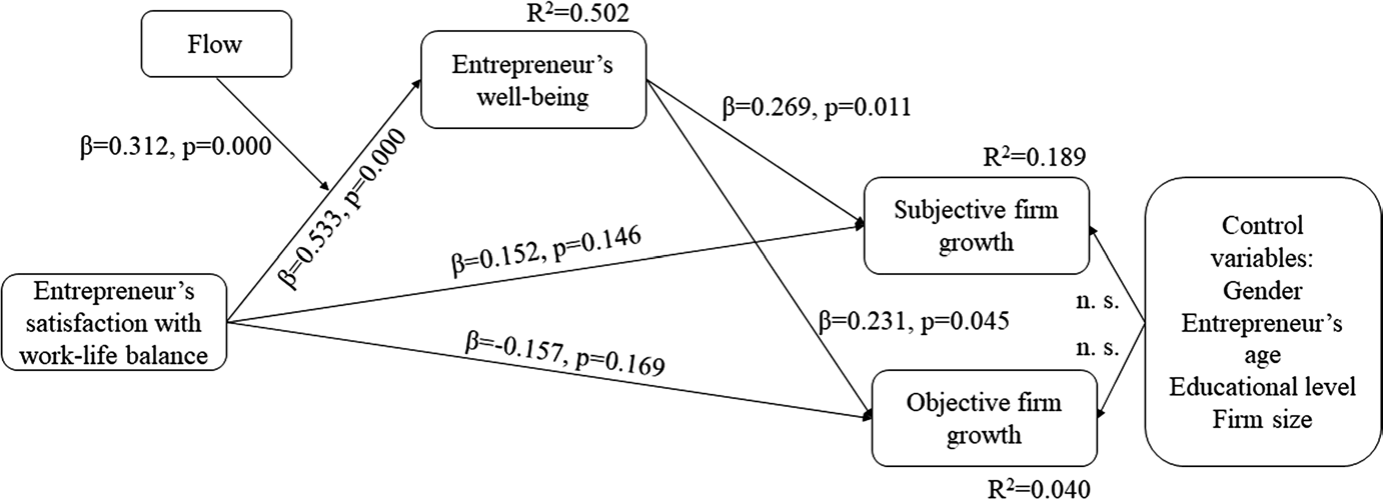
Results for the moderated mediation model. *Note* n. s. stands for non-significant effect

**Fig. 3:**
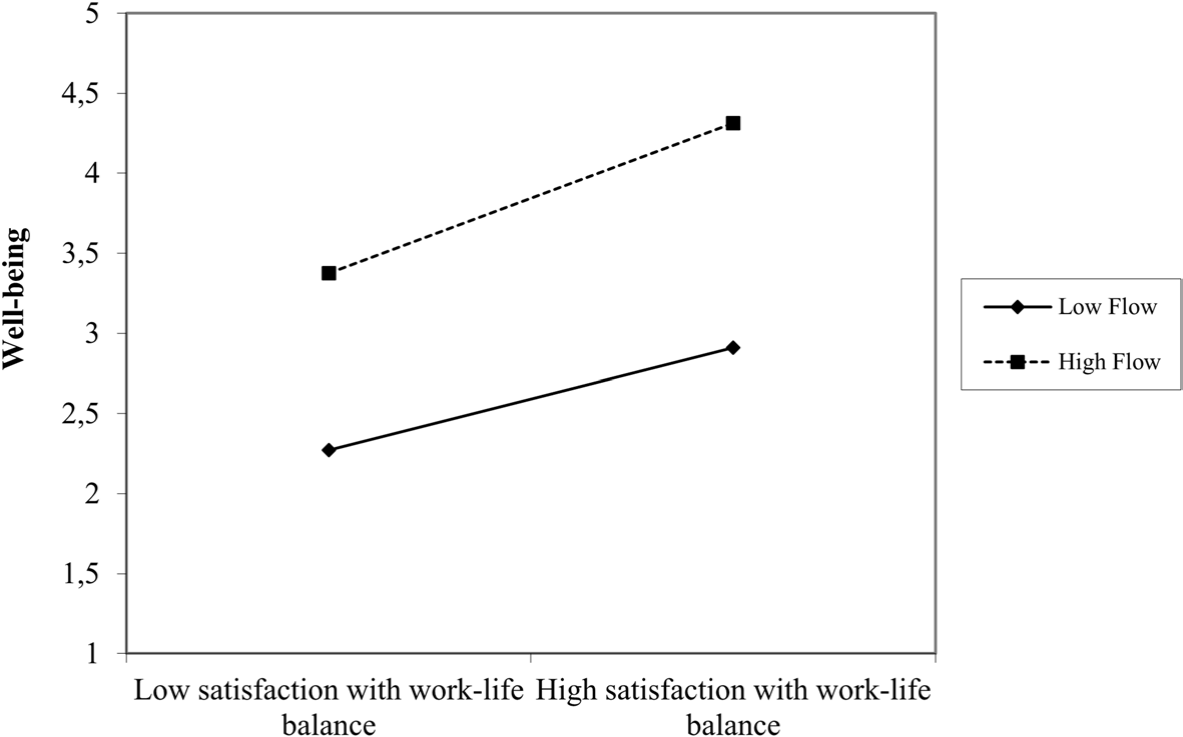
Moderation effect of flow on the relationship between satisfaction with work-life balance and well-being

Finally, we tested the moderation effects of flow at work on the relationship between entrepreneurs’ satisfaction with work–life balance and well-being by introducing the interaction term (flow at work x satisfaction with work–life balance) into our model (see H4 in Table 3). The results show that flow at work is a significant moderator in the relationship between satisfaction with work–life balance and subjective well-being such that higher levels of flow at work enhance subjective well-being when satisfaction with work–life balance is high. These findings support Hypothesis 4 (β = 0.312, *p* = 0.000), and model fit was adequate: χ^2^ = 33.001, df = 25, *p* = 0.131, CFI = 0.937, RMSEA = 0.053, and SRMR = 0.078. Figure 2 summarizes the results of the moderated mediation model. Figure 3 presents the moderation effect of flow on the relationship between entrepreneurs’ satisfaction with work–life balance and well-being.

## Discussion and conclusion

### Theoretical implications

This study contributes to the literature on entrepreneurs’ characteristics and firm growth by integrating the theoretical perspective of COR theory. Aligned with COR theoretical predictions, we support the premise that satisfaction with work–life balance and experience of flow at work are important personal resources that entrepreneurs use to protect their valued outcomes, such as subjective well-being. Specifically, the results of our study suggest that an entrepreneur’s satisfaction with work–life balance is positively associated with firm growth, and this association is mediated by the entrepreneur’s subjective well-being. According to COR theory, subjective well-being can be perceived as a desired personal outcome that contributes to the resource gain spiral to support firm-level outcomes. We emphasize the benefits of achieving satisfaction with work–life balance in an entrepreneurial career because it is important for an entrepreneur’s subjective well-being and has a positive impact on firm growth. These findings may shift the attitudes of entrepreneurs and other important stakeholders in entrepreneurial ecosystems toward embracing subjective well-being as an important indicator of organizational performance alongside traditional financial measures.

First, we contribute to the development of research on entrepreneurs’ well-being by demonstrating new antecedents of entrepreneurs’ subjective well-being as an important personal-level outcome in entrepreneurship, thus expanding its nomological network. Although some previous studies (for example, Cooper and Artz (1995)) have underlined the importance of entrepreneurs’ personal satisfaction, the majority of existing studies have used firm-level outcomes such as growth as key performance indicators and relevant indicators of entrepreneurial success (Wiklund et al. 2019). We go beyond existing studies by making a theoretical and empirical attempt to establish a critical link between individual and organizational outcomes in entrepreneurship, namely, between entrepreneurs’ subjective well-being and firm growth. We draw from COR theory to conceptualize and empirically test why it is important for entrepreneurs to nurture their work–life balance and well-being as key personal resources facilitating firm growth. In doing so, we contribute to the existing body of literature on entrepreneurs’ well-being by theorizing that subjective well-being as a desired outcome critically contributes to entrepreneurs’ accumulation of a personal resource reservoir by initiating resource gain spirals. Although existing evidence suggests that individual factors are determinants of firm growth (Sarwoko and Frisdiantara 2016), little research has examined how individual factors interact to promote firm growth. Our findings emphasize that satisfaction with work–life balance is most likely to be associated with higher well-being when entrepreneurs also experience flow at work, which, in turn, generates more resources that entrepreneurs can harness to increase subjectively perceived firm growth. Our study suggests that entrepreneurs who have accumulated sufficient personal resources (i.e., satisfaction with work–life balance, experience of flow at work and well-being) are more likely to grow their firm. Interestingly, in contrast to previous studies that have suggested a strong positive correlation between subjective and objective firm performance, thereby justifying the use of subjective measures of firm performance (Vij and Bedi 2016), our study suggests that personal resources positively influence subjective firm growth only. To further expand theoretical perspectives in the growing body of research on entrepeneurial well-being, the COR perspective we use in this study invites consideration of other resources that entrepreneurs avail: organizational social resources, and job-related, home and personal resources (Lee et al. 2020). While we focused on personal resources within one’s psychological sphere in this study, future studies could consider the interplay between different types of resources within the personal sphere of entrepreneurs. Previous research has emphasized the importance of human and social capital (Chandler and Hanks 1998) for growing and developing firms. It would therefore be interesting to examine how access to these types of resources impacts subjective well-being and contributes to the accumulation of personal resource reservoirs. Our findings also invite scholars to further examine how personal resources interact with other types of resources to influence subjective and objective firm growth, and to explore the interdependence between subjective and objective firm growth.

We also contribute to the stream of literature on work–life balance by providing insights into work–life balance experiences in smaller organizations (Lingard et al. 2015) and taking a step toward understanding its impact on personal and organizational outcomes. In line with the main premises of COR theory, we proposed and found that satisfaction with work–life balance serves as a personal resource that is positively associated with both entrepreneurial well-being (i.e., personal outcome) and subjective firm growth (i.e., organizational outcome). Our findings are consistent with existing literature suggesting that individuals who are able to maintain a satisfactory work–life balance experience less work–life conflict and stress, have a higher quality of life and well-being, and are more prone to growth and development within work and non-work domains (Greenhaus et al. 2003; Cegarra-Leiva et al. 2012; Kalliath and Brough 2008). Moreover, our study demonstrates the direct relationship between entrepreneurs’ satisfaction with work–life balance and subjective firm growth, thereby lending credibility to the assumption that non-financial criteria are important for measuring business success and are significantly related to financial indicators. We also make an important contribution to the work–life balance literature by providing empirical evidence on the regulatory role of the flow experience at work in amplifying the effects of satisfaction with work–life balance on the subjective perception of well-being. Our results suggest that entrepreneurs who experience high levels of satisfaction with work–life balance and flow at work are more likely to successfully achieve their personal outcomes and are thus more inclined to grow their business.

Finally, we contribute to COR theory, since our conceptual framework proposes that entrepreneurs’ satisfaction with work–life balance, experience of flow at work and subjective well-being are important resources for enterprising individuals. Accordingly, we support the core tenet of COR theory that family and well-being are among the most valued resources of individuals. Given that our empirical dataset consists of subjective and objective data, we are able test the specific theoretical mechanisms postulating that entrepreneurs can assimilate and invest resources in resource gain spirals that supplement organizational outcomes, such as firm growth.

### Practical implications

Our findings make several practical contributions. First, they foreground the critical nature of entrepreneurs’ personal resources, which has become even more pronounced during the global health crisis induced by the COVID-19 pandemic (Sharma et al. 2022) and the disruptions caused by the unprecedented effects of climate change and the Russian war in Ukraine (Bouncken et al. 2022). These disruptions have presented challenges in all spheres of entrepreneurial functioning (Afshan et al. 2021; Emami et al. 2022) by altering approaches to entrepreneurial strategy making (Rapp 2022) and creating additional strain, making it difficult to maintain adequate levels of personal resources. Fortunately, our research sheds light on the coping mechanisms associated with resource gains. We find that entrepreneurs who experience high levels of flow at work also experience higher levels of subjective well-being; this, in turn, may sustain and/or increase organizational outcomes, such as firm growth. Entrepreneurship is likely the work environment most conducive to experiencing flow, and entrepreneurs continue to start new businesses as much for the flow experience as for the additional success (Sawyer 2017). However, entrepreneurs cannot continuously experience flow when dealing with the same challenges (Csikszentmihalyi 1991). To sustain firm growth, therefore, we recommend that entrepreneurs search for and create challenging work environments, and persistently improve their entrepreneurial skill level with the goal of continuously experiencing flow at work. Additionally, our findings highlight the importance of achieving a satisfying work–life balance, as its direct and indirect interplay with the experience of flow at work contributes to entrepreneurs’ well-being as a desired personal outcome. Although entrepreneurs often face the challenge of maintaining the right work–life balance (Gröpel and Kuhl 2006), our results suggest that those entrepreneurs who can overcome this challenge will experience desired individual and organizational goals.

We suggest that to improve organizationally relevant outcomes (e.g., firm growth), entrepreneurs should consistently monitor their subjective satisfaction with work–life balance and organize their work in a way that enables them to master that balance. This can be achieved, for example, by creating a daily routine that helps them make time for things that are important to them, setting boundaries between work and personal time, taking time off, and learning to prioritize. In doing so, they will not only build and conserve resources to respond to existing strain, but also initiate resource gain spirals to build a reservoir of sustaining resources to meet potential needs. Personal resources vitally support entrepreneurial behaviors when entrepreneurs face uncertainties in managing multiple roles at once, such as work and family roles. Therefore, entrepreneurs need to protect against resource loss and develop skills to orchestrate resource gains.

### Limitations and future research avenues

As with any research, there are some limitations related to our study. First, we have partly relied on self-reported data, as we were interested in studying entrepreneurs’ subjective experiences of work–life balance, well-being, and flow at work, similar to other studies in this domain (Wach et al. 2021). Although many authors have pointed out the limitations of self-reported data (see Robins et al. 2007), there are certain advantages to this design that are relevant to our research. For example, self-ratings are the best way to capture the subjective attitudes and perceptions of participants (Parker and Collins 2010). Furthermore, some researchers have argued (see Evans 1985) that the relationships hypothesized among variables usually have complex interactions that cannot be attributed to common method variance effects. Acknowledging the potential limitations of this approach, we have used several procedures and statistical remedies to minimize and control for common method bias, as reported in the results section of the paper. Nevertheless, as with any survey design, the possibility of common method variance bias remains. We should also note that entrepreneurship is not an idyllic process and that some reverse effects might be in place. For example, entrepreneurs who experience lower rates of firm growth or even losses might, in turn, experience lower satisfaction with their work–life balance and lower subjective well-being, especially considering that entrepreneurs often become intertwined with their ventures and experience firm losses as their own losses or own low performance (Cardon et al. 2009; Pierce et al. 2001).

Second, our data were collected within one national context, so it could be argued that specific cultural and national factors may have affected the results. In response, we reason that cultural/national factors should not play a significant role in our model’s empirical results, based on the literature examining the impact of changing national contexts on work–life experiences (Trefalt et al. 2013). In terms of the subjective perceptions of work–life balance and well-being, previous research has suggested that people tend to compare their individual work–life experiences and well-being over time to those of their family members, friends and peers. As in the case of our research model, these findings suggest that any changes and variations associated with national and cultural factors would affect all individuals in a similar (relative) manner. However, this is not to say that understanding the impact of cultural factors is not important. Acknowledging the role of culture, we suggest that future research could explore the specific mechanisms through which cultural and national contexts impact an individual’s evaluations of work–life balance and well-being, and the significance of that impact.

Third, there may be some concerns associated with the validity of the constructs used in our survey. Csikszentmihalyi (1990) himself acknowledged the interconnection between his concept of flow and many other concepts, such as engagement, involvement, passion, thriving, intrinsic motivation and peak experience. As reported in the results section, we performed several measurement tests to assess the reliability and validity of the constructs used in the study. Although we measured entrepreneurs’ experience of flow at work at the individual level, an interesting avenue for future research could be related to team-level flow, considering that flow may also be experienced in interactions with friends, co-workers and family (Csikszentmihalyi 1990; Csikszentmihalyi & LeFevre 1989). Indeed, social flow may be more enjoyable than solitary flow. Since the results support the impact of flow on the growth of small and medium-sized enterprises, future research is needed to examine the influence of team-level flow (the flow experience of entrepreneurs and their co-workers or in an entrepreneurial team) on important firm growth-related variables.

### Conclusion

In this study, we maintain that the personal resources of entrepreneurs (i.e., satisfaction with work–life balance, subjective well-being and experience of flow at work) are crucial for entrepreneurs because these resources can have a positive impact on society through their influence on firm growth. Drawing from COR theory, we postulate testable hypotheses to empirically test the importance of achieving satisfactory work–life balance due to its effects on subjective well-being and firm growth. We find empirical support for these hypotheses. We also find support for the resource gain spiral that accentuates the effects of the flow experience at work.

## Supplementary Information

Below is the link to the electronic supplementary material.

Supplementary file1 (DOCX 25 kb)
