# Supplementary material for: “I want it all”: exploring the relationship between entrepreneurs’ satisfaction with work–life balance, well-being, flow and firm growth
Source: Rev Manag Sci. 2023 Mar 2:1–28. Online ahead of print. doi: 10.1007/s11846-023-00623-2 (PMC9979118; doi:10.1007/s11846-023-00623-2)
Supplement: Supplementary file 1 — Supplementary file1 (DOCX 25 kb) [file 11846_2023_623_MOESM1_ESM.docx]

Appendix:

Table A1: Measurement scales and standardized factor loadings of items from confirmatory factor analysis

| **Scale** | **Factor loading** |
| --- | --- |
| **Work-life balance satisfaction**  Putrevu &Ratchford (1997)  (1=Very dissatisfied to 5=Very satisfied)  I am satisfied with… |  |
| … the way I divide my time between work and non-work life. | 0.807 |
| … the way I divide my attention between work and non-work life. | 0.817 |
| … how well my work life and my non-work life fit together. | 0.879 |
| … my ability to balance the needs of my job with those of my non-work life. | 0.884 |
| … the opportunity I have to perform my job well and yet be able to perform nonwork related duties adequately. | 0.860 |
| **Entrepreneur’s subjective well-being**  Su, Swanson, & Chen (2016)  (1=Strongly disagree to 5=Strongly agree) |  |
| In general, I consider myself a very happy person. | 0.712 |
| Compared to most of my peers, I consider myself more happy. | 0.649 |
| I am generally very happy and enjoy life. | 0.832 |
| **Flow at work**  Mao, Roberts, Pagliaro, Csikszentmihalyi, & Bonaiuto, (2016)  (1=Strongly disagree to 5=Strongly agree)  Think of an activity, which you like to do at work and mark the level of agreement about the following statements.  When I engage in this activity… |  |
| I feel in control. | 0.507 |
| I feel I know how well I am doing. | 0.413 |
| I have a high level of concentration. | 0.635 |
| I forget about personal problems. | 0.495 |
| I feel fully involved. | 0.770 |
| **Subjective firm growth**  Adapted from Dijkhuizen, Gorgievski, van Veldhoven, & Schalk, (2018)  (1=much worse than competitors to 5=much better than competitors)  In the last three years our company has been better/worse compared to competitors in our industry at… |  |
| … revenue growth. | 0.919 |
| … growth in the number of employees. | 0.734 |
| … growth in market share. | 0.912 |
| **Objective firm growth** |  |
| Revenue growth of each company in the period 2017-2020 divided by the average revenue growth of the industry in the same period | **/** |
